# Performance of EQ-5D, *howRu* and Oxford hip & knee scores in assessing the outcome of hip and knee replacements

**DOI:** 10.1186/s12913-016-1759-x

**Published:** 2016-09-22

**Authors:** Tim Benson, Dan H Williams, Henry W W Potts

**Affiliations:** 1 R-Outcomes Ltd, Hermitage, Thatcham, RG18 9WL UK; 2 UCL Institute of Health Informatics, 222 Euston Road, London, NW1 2DA UK; 3 Royal Cornwall Hospital, Truro, TR1 3LJ UK; 4 MyClinicalOutcomes Ltd, London, UK

**Keywords:** Patient-reported outcome measures, Total hip arthroplasty, Total knee arthroplasty, EQ-5D, howRu, Oxford hip score, Oxford knee Score

## Abstract

**Background:**

We aimed to compare the performance of EQ-5D-3 L and *howRu*, which are short generic patient-reported outcome measures (PROMs), in assessing the outcome of hip and knee replacements, using the Oxford Hip Score (OHS) and the Oxford Knee Scores (OKS) for comparison.

**Methods:**

Outcome was assessed as the difference between pre-surgery and 6-month post-surgery scores. We used a large sample from the NHS PROMs database, which used EQ-5D-3 L, and a small cohort of patients having the same operations collected by MyClinicalOutcomes (MCO), which used *howRu*. Both cohorts completed the OHS (hips) or the OKS (knees).

**Results:**

The change (outcome) between pre-op and post-op scores as measured by *howRu* was greater than that measured by EQ-5D, relative to that measured by OHS or OKS.

For hip replacements, the correlation for change measured by *howRu* and OHS was *r* = 0.77 (0.66–0.85). The corresponding correlation for change measured by EQ-5D Index and OHS was *r* = 0.64 (0.63–0.64).

For knee replacements the correlation between change in *howRu* and OKS was *r* = 0.86 (0.75–0.92); between EQ-5D Index and OKS *r* = 0.59 (0.58–0.60).

**Conclusions:**

For hip and knee replacement, the outcome measured by *howRu* was more highly correlated with that measured by the condition-specific Oxford Hip and Knee Scores than were EQ-5D Index or EQ-VAS. The magnitude of change before and after surgery was also greater.

**Electronic supplementary material:**

The online version of this article (doi:10.1186/s12913-016-1759-x) contains supplementary material, which is available to authorized users.

## Background

Patient-reported outcome measures (PROMs) have the potential to help improve health and care services by focusing attention on what matters most to people receiving treatment [1–3].

Changes measured using different measures should be highly correlated and show similar change magnitude. Many different measures have been developed but the changes measured by different instruments do not agree well [4]. Direct comparisons between two measures show the extent of agreement between them, but cannot show whether one measure is better than another. For this we need a gold standard for comparison.

In this study, we set out to compare the changes following hip and knee replacement surgery as measured by two generic PROMs – EQ-5D-3 L [5] and *howRu* [6] – using condition-specific measures – Oxford Hip Score (OHS) [7] and Oxford Knee Score (OKS) [8] – for comparison.

We compared comparable cohorts from two existing databases as a natural experiment – NHS PROMs and MyClinicalOutcomes. Since 2009, all patients having hip and knee replacement surgery paid for by the NHS have been asked to complete EQ-5D-3 L and the Oxford scores before and six months after surgery. Anonymised results are published for further analysis. This programme has led to more than 60 research papers [9]. MyClinicalOutcomes has collected a database on a wide range of patients where it has collected *howRu* and the Oxford Scores [10]. We extracted a subset of those with hip and knee replacement surgery. This allows a comparison of EQ-5D-3 L with *howRu* by seeing how both perform against the same condition-specific measures on similar cohorts of patients.

### The measures

The OHS [7] and the OKS [8] are condition-specific PROMs for the evaluation of joint replacement implants and techniques. Each measure has 12 items, with five responses each. Each item is scored on a 0–4 scale. The score for each item is added, giving an overall score on a scale from 0 (worst possible score) to 48 (best possible score).

EQ-5D-3 L [5] is a generic PROM with two parts, the EQ-5D Index and a visual analogue scale (EQ-VAS). The EQ-5D Index is derived from 5 items: mobility (walking about), self-care (washing and dressing), usual activities (e.g. work, study, housework, family or leisure activities), pain or discomfort, and anxiety or depression. Each item has 3 possible responses. The EQ-5D Index is derived by applying weights to each response based on valuations derived from a population survey. The NHS PROMs programme uses the UK tariff [11]. These weights purport to represent the perspective of society as a whole. The range of possible scores for the EQ-5D Index is from −0.594 (worst state) to 1.0 (best state), with death allocated a value of 0. The EQ-VAS is a 20 cm visual analogue scale with a range from 0 (worst imaginable health state) to 100 (best imaginable health state). The EQ-VAS is intended for use as a quantitative measure of health outcome as judged by the individual respondents [12, 13].

*HowRu* [9] is a short generic patient-reported measure of health-related quality of life, with 4 items: pain or discomfort; feeling low or worried; limited in what you can do; need help from others. Each item has four possible responses: extreme, quite a lot, a little, and none. These are scored from 0 (extreme) to 3 (none). The summary *howRu* score is the sum of the item scores, giving a scale with 13 possible values with a range from 0 (4 × extreme) to 12 (4 × none).

**Fig. 1:**
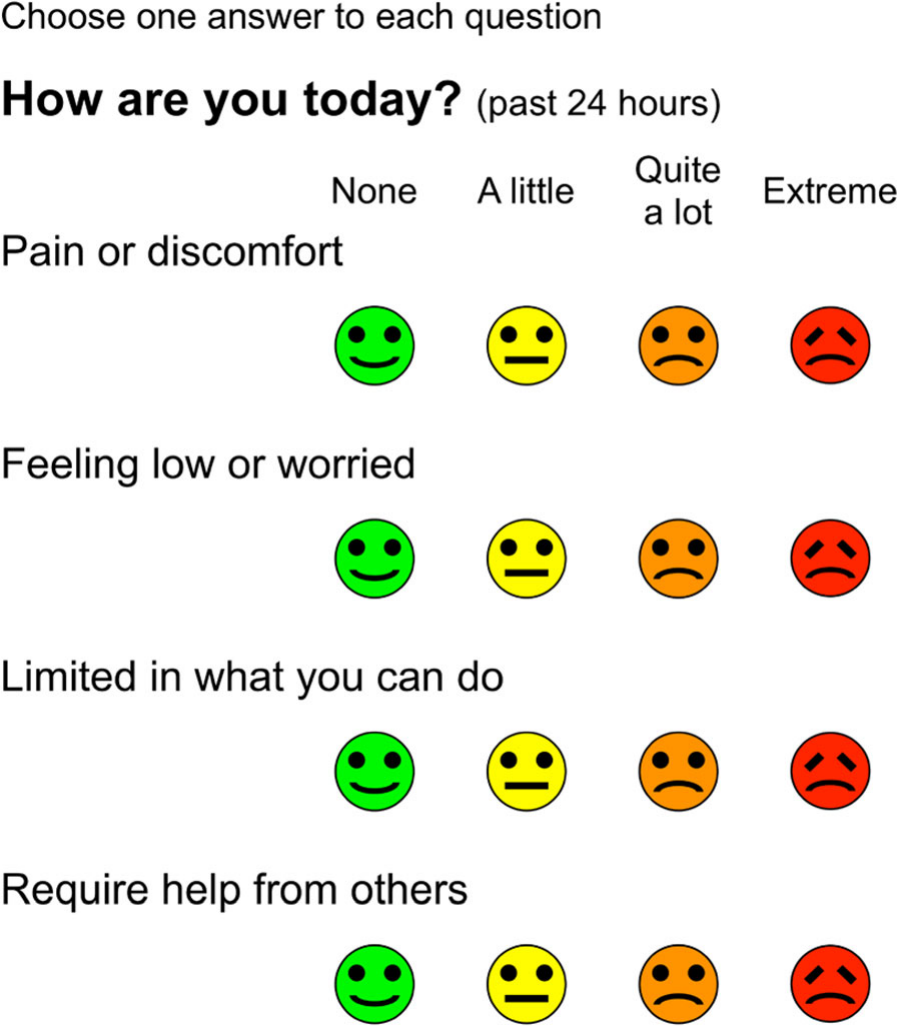
*howRu* instrument

Previous studies have compared *howRu* with SF12 [6] and with EQ-5D [14], and show that *howRu* has comparable overall performance at a single point in time. *HowRu* is considerably shorter than EQ-5D with 37 words vs. 230 words and has been validated for use at the individual patient level [15]. Since the original publication of *howRu* [6], some small changes have been made. The original item "Dependent on others" has been changed to "Require help from others", to improve understanding. The user instructions have been simplified from "Circle one face on each line to tell us how you are today" to "Choose one answer to each question". The main question "How are you today?" has been qualified by adding "(past 24 hours)" to clarify that it means this day rather than right now. These changes have slightly changed the word counts (see Fig. 1).

All of these instruments were developed as measures of patient benefit, so we might expect that they would show a similar level of improvement and be highly correlated. However, condition-specific measures only take account of those aspects of each patient’s health directly associated with the condition being treated, while generic measures have a more holistic view, including co-morbidities. For this reason, condition-specific measures usually show larger improvements after surgery than generic measures [3].

## Methods

The data collected in the NHS PROMs programme covers all hospitals providing hip and knee replacements paid by the NHS. Most data are collected using paper booklets. Pre-operative questionnaires are completed at a pre-operative assessment clinic or on admission. Post-operative questionnaires are mailed to each patient’s home address 6 months later.

To use the MCO web-based system, patients register, complete the appropriate condition-specific measures (here, OHS or OKS) and *howRu*, and consent to share their health information with their medical team. Patients are issued new question sets every three months and are shown feedback indicating the absolute and rate of change in their score. The MCO data for this study was collected between August 2011 and October 2013. The MCO data is not publicly available.

The MCO system had 1,696 patients with an OHS and 1,395 patients with an OKS. Of these, 178 hip replacement patients and 103 knee replacement patients had both a pre-operative and post-operative ratings. The proportion is relatively low because most patients also completed NHS PROMs surveys for hip and knee replacement operations, which involved duplication of the OHS and OKS scores. Entries with matched pre-op and a 5, 6 or 7-month post-op ratings for both *howRu* and OHS or OKS as appropriate were selected for analysis. Where more than one set of post-operation ratings was available, we selected the one closest to 6 months after the operation. All patient records that were incomplete for any reason were excluded from the analysis. This yielded data on 74 hip replacements and 42 knee replacements.

The original scores for both NHS and MCO records were used without case-mix adjustment.

Each instrument uses a different scale, which complicates comparison between results using different instruments (Table 1). We transformed each scale arithmetically to provide a common 0–100 scale from minimum (0) to maximum (100).

**Table 1:** Range of possible scores for each instrument

Measure	Use	Minimum score	Maximum score
OHS	Hip replacement	0	48
OKS	Knee replacement	0	48
EQ-5D Index	Generic	−0.594	1.0
EQ-VAS	Generic	0	100
*HowRu*	Generic	0	12

We used Excel or Stata/IC for Windows 12.1 to calculate the distributions, means, standard deviations and correlations for each measure.

The generic measures are compared with condition-specific measures in the following ways.

- The proportion of patients reporting improvements using each measure.
- Pre-op and post-op scores for each measure.
- The mean change between each patient’s pre-operative and post-operative scores for each measure, using the 0–100 scale.
- Correlation of the change between pre-operative and post-operative scores for each generic measure with the relevant condition-specific measure.

## Results

Table 2 shows the number of patients in each cohort and the proportion of patients who have shown improvement for each measure with the 95 % confidence limits.

**Table 2:** Percentage of patients reporting any improvement and 95 % confidence intervals

	# Improved	% Improved	95 % Confidence Intervals
Hip Replacements			
* NHS PROMs* (*n = 29,129*)			
OHS	28023/29129	96.2 %	96.0 %–96.4 %
EQ-5D Index	25572/29129	87.8 %	87.4 %–88.2 %
EQ-VAS	18716/29129	64.3 %	63.7 %–64.8 %
* MCO* (*n = 74*)			
OHS	71/74	96.0 %	88.6 %–99.2 %
* HowRu*	68/74	91.9 %	83.2 %–97.0 %
Knee Replacements			
* NHS PROMs* (*n = 29,907*)			
OKS	27633/29907	92.4 %	92.1 %–92.7 %
EQ-5D Index	23657/29907	79.1 %	78.6 %–79.6 %
EQ-VAS	16204/29907	54.2 %	53.6 %–54.8 %
* MCO* (*n = 42*)			
OKS	42/42	100.0 %	91.6 %–100 %
* HowRu*	33/42	78.6 %	63.2 %–89.7 %

Table 3 shows, for each cohort and measure, the mean pre-operative and post-operative scores and the mean change after surgery (the outcome), calculated as the post-operative score minus the pre-operative score. These are shown transformed to a common 0–100 scale. The same data using the original scales are provided as an Additional file 1.

**Table 3:** Mean pre-op and post-op scores and the mean change after surgery (post-op score minus pre-op score) using 0–100 scales

0–100 scale mean			
	Pre-op score	Post-op score	Change after surgery (Post-op minus Pre-op)
Hip Replacements			
* NHS PROMs* (*n = 29,129*)			
OHS	38.0	80.1	42.2
EQ-5D Index	59.7	85.7	26.0
EQ-VAS	65.4	75.6	10.2
* MCO* (*n = 74*)			
OHS	41.6	85.4	43.9
* HowRu*	55.2	87.7	32.5
Knee Replacements			
* NHS PROMs* (*n = 29,907*)			
OKS	39.4	71.2	31.8
EQ-5D Index	62.9	81.8	18.9
EQ-VAS	67.6	72.2	4.6
* MCO Knee Replacements* (*n = 42*)			
OKS	40.3	76.7	36.4
* HowRu*	56.5	82.1	25.6

**Fig. 2:**
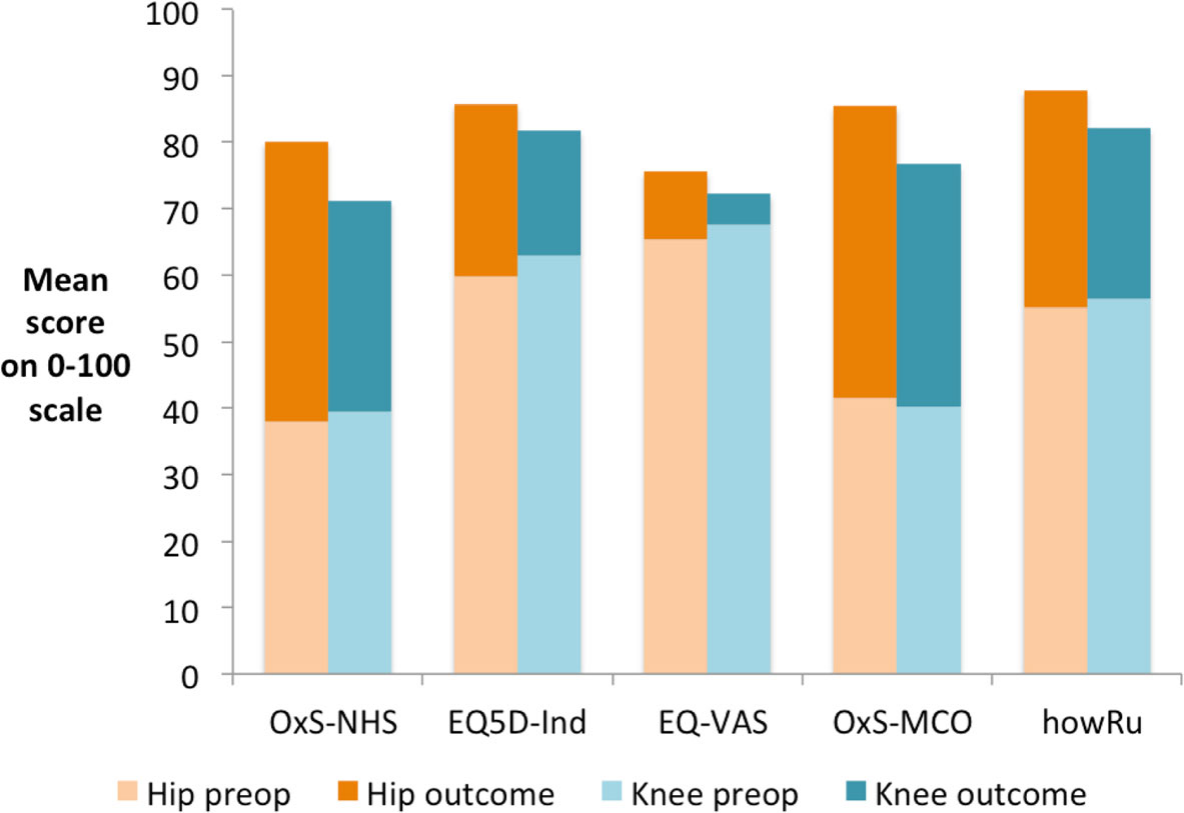
Relative size of scores before and six months after hip and knee replacement surgery as measured by different instruments on a common 0–100 axis, where 0 represents the worst state on each scale and 100 represents the best possible score. OxS refers to Oxford Hip Score for hip replacement and Oxford Knee Score for knee replacement; EQ5D-Ind refers to the EQ-5D Index score and EQ-VAS the EQ-5D Visual Analogue Score. OxS-MCO and howRu scores are from MyClinicalOutcomes data, others are from the NHS PROMs data

The use of the 0–100 scale allows a comparison of the outcome as measured by each instrument for each type of operation (Fig. 2). For hip replacement, EQ-5D shows an improvement of 26.0, compared with 42.2 for OHS (62 % of the OHS score) for the NHS cohort. *HowRu* shows an improvement of 32.5 compared with 43.9 for OHS (74 %) for the MCO cohort.

For knee replacement, EQ-5D shows an improvement of 18.9, compared with 31.8 for OKS (59 %) for the NHS cohort. *HowRu* shows an improvement of 25.6 compared with 36.4 for OHS (70 %) for the MCO cohort.

The MCO patients have greater improvement than the NHS patients, which may be due to different populations. The *howRu* instrument shows a greater improvement, relative to the condition-specific measure than EQ-5D.

**Fig. 3:**
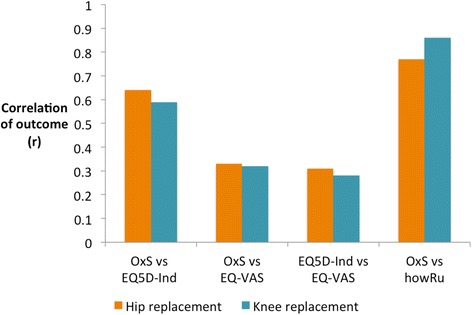
Correlations of the outcome of hip and knee replacement surgery as measured by Oxford Hip and Knee Scores (OxS), EQ-5D Index, EQ-VAS (for NHS PROMs data) and howRu (for MCO data)

The correlations for each measure within each cohort are shown in Table 4 for the scores before surgery and 6 months after surgery. Table 5 and Fig. 3 show the correlation of the change or outcome of surgery, as measured by each instrument.

**Table 4:** Correlations between condition-specific and generic scores

	Pre-op (95 % confidence interval)	Post-op (95 % confidence interval)	Pre-op *z*-test compared to correlation with *howRu*	Post-op *z*-test compared to correlation with *howRu*
Hip Replacement				
OHS vs. *howRu* (MCO)	0.82 (0.73–0.86)	0.80 (0.75–0.84)		
OHS vs. EQ-5D Index	0.74 (0.74–0.74)	0.76 (0.76–0.76)	*z* = 1.82, *p* = 0.069	*z* = 0.84, *p* = 0.4
OHS vs. EQ-VAS	0.38 (0.37–0.39)	0.60 (0.60–0.61)	*z* = 6.45, *p* < 0.0001	*z* = 3.37, *p* = 0.0008
EQ-5D Index vs. EQ-VAS	0.36 (0.35–0.37)	0.64 (0.64–0.64)		
Knee Replacement				
OKS vs. *howRu* (MCO)	0.84 (0.71–0.88)	0.79 (0.72–0.84)		
OKS vs. EQ-5D Index	0.70 (0.70–0.70)	0.77 (0.77–0.77)	*z* = 2.10, *p* = 0.036	*z* = 0.34, *p* = 0.7
OKS vs. EQ-VAS	0.38 (0.37–0.38)	0.60 (0.59–0.60)	*z* = 5.05, *p* < 0.0001	*z* = 2.39, *p* = 0.017
EQ-5D Index vs. EQ-VAS	0.35 (0.34–0.36)	0.62 (0.62–0.63)

**Table 5:** Correlations of differences between post-op and pre-op scores

	Difference (95 % confidence interval)	*z*-test compared to correlation with *howRu*
Hip Replacement		
OHS vs. *howRu* (MCO)	0.77 (0.66–0.85)	
OHS vs. EQ-5D Index	0.64 (0.63–0.64)	*z* = 2.36, *p* = 0.018
OHS vs. EQ-VAS	0.33 (0.32–0.34)	*z* = 5.77, *p* < 0.0001
EQ-5D Index vs. EQ-VAS	0.31 (0.30–0.32)	
Knee Replacement		
OKS vs. *howRu* (MCO)	0.86 (0.75–0.92)	
OKS vs. EQ-5D Index	0.59 (0.58–0.60)	*z* = 3.86, *p* = 0.0001
OKS vs. EQ-VAS	0.32 (0.31–0.33)	*z* = 6.02, *p* < 0.0001
EQ-5D Index vs. EQ-VAS	0.28 (0.27–0.29)

The correlations between *howRu* with OHS and OKS are higher than the corresponding correlations with EQ-5D Index. Tables 4 and 5 also give *z*-tests comparing the correlations: correlations with *howRu* are statistically significantly higher than with EQ-5D Index for the outcome of hip and knee replacement and pre-operatively for the knee replacement. The correlations of OHS and OKS with *howRu* are much higher and statistically significantly higher than those with the EQ-VAS. For example, considering the outcomes of hip surgery, a correlation of *r* = 0.77 (OHS vs. howRu) explains 59 % of the variance (r^2^), while correlation of *r* = 0.64 (OHS vs. EQ-5D Index) explains 41 % of the variance and correlation of *r* = 0.33 (OHS vs. EQ-VAS) explains only 11 % of the variance.

## Discussion

In a previous paper, [14] we compared and discussed the differences between *howRu* and EQ-5D in a study of the same population. That study showed that *howRu* is shorter, has better readability statistics, a higher completion rate, used a wider range of states and has a smaller ceiling effect than EQ-5D.

This study suggests that, for similar types of patient, *howRu* shows larger relative improvements, compared with condition-specific measures, than the EQ-5D Index and much larger improvements that EQ-VAS. *HowRu* also shows higher correlations for the surgery outcome, the difference between pre and post-operative scores.

One explanation for these differences may be the noise introduced by the weighting system or tariff used to calculate the EQ-5D Index scores. This view is supported by the release of the new tariff for EQ-5D-5 L [16], which has substantial differences from that used for the 3 L version [17].

The scores calculated in this paper for NHS patients, covering a 6-month period without risk adjustment, are very similar to those presented in the final published results for the whole year 2011–12, which include risk adjustment [18].

The condition-specific scores show high levels of improvement (the means are between 31.8 and 43.9 on the 0–100 scale). Generic measures such as EQ-5D Index and *howRu* capture each patient’s symptoms and disability from any cause (not just hips or knees). These show substantial but not as high improvements (between 18.9 to 32.5). On all measures, the results at six months are better for hips than for knees.

The improvements measured by EQ-VAS (10.2 for hips and 4.6 for knees) are much lower than for EQ-5D Index. EQ-VAS also shows low correlations with the EQ-5D Index. These large differences between EQ-VAS and EQ-5D Index were known in the 1990s for patients with rheumatic disease such as those having hip and knee replacement [19]. The new EQ-5D-5 L version [16] with more response levels may have better properties [20].

Feng, Parkin and Devlin [21] investigated the performance of the EQ-VAS in the NHS PROMs programme with similar results to those presented here and suggested that the results might be improved by providing better guidance on collection and coding. Our view is that EQ-VAS is measuring something substantially different from the other measures. EQ-VAS asks the patient to rate their health state on a scale with end points of best and worst imaginable health states. This implies inclusion of aspects such as prognosis (including that of other comorbidities), social deprivation and optimism, which are not covered by the other measures and may not be changed by joint replacement.

Hip and knee replacements are major operations with substantial costs in terms of both money and post-operative recovery periods. For these, and indeed all operations, patients, surgeons and commissioners need to know the likelihood of a favorable outcome. However, preliminary analysis of the first three years results of the NHS PROMs programme has shown little impact on hospital performance [22]. This may in part be because information feedback was slow. For example, the final results for operations performed in 2009 were not released until August 2011. Furthermore, these results were issued using a complex interactive spreadsheet (the PROMs Score Comparison Tool) [23] that is difficult to use.

Each measure uses a different scale range, which creates a barrier to comparison and understanding [24]. Transformed 0–100 scales, shown in Table 3 and Fig. 2, are much easier to interpret than the original scales when comparing mean scores. To illustrate this, Table 6 shows the original and the 0–100 scales for the average change as measured by the Oxford scores, EQ-5D and EQ-VAS for NHS hip and knee replacements. The original scales are shown in the Additional file 1.

**Table 6:** Comparison of mean improvement between pre-op and post-op measure on the original and transformed (0–100) scales

Improvement	Original scales	0–100 scale
NHS hip replacements		
OHS	20.2	42.2
EQ-5D Index	0.415	26.0
EQ-VAS	10.2	10.2
NHS knee replacements		
OHS	15.3	31.8
EQ-5D Index	0.301	18.6
EQ-VAS	4.6	4.6
MCO hip replacements		
OHS	21.1	43.9
* howRu*	3.9	32.5
MCO Knee replacements		
OKS	17.5	36.4
* howRu*	3.1	25.6

Limitations of this study include the modest number of MCO patients analysed. However, confidence intervals show that the numbers are statistically precise enough for our purposes. Case-mix adjustment was not applied to the scores [25]. The mean pre-operative condition-specific scores for the MCO cohorts are not significantly different from the NHS scores, but the postoperative scores are higher than the corresponding NHS scores (*p* < 0.05). This may be because the MCO patients comprise a different population from the NHS group, being younger [26], less deprived [27], more self-selecting and self-motivated [28], all of which may contribute to better outcome. NHS patients may have more co-morbidity, which might increase the gap between condition-specific and generic outcomes.

## Conclusions

In this study, *howRu*, as a generic score, better measures improvement following hip and knee replacement surgery than EQ-5D compared to the OHS/OKS gold standard. Given the wide use of EQ-5D, we recommend that larger studies confirm or refute these findings.

## Additional file

Additional file 1: Summary statistics scores using original scales. (DOCX 16 kb)

## Abbreviations

EQ-5D: EuroQol 5 dimensions
EQ-VAS: EuroQol visual analogue scale
HSCIC: Health and social care information centre
MCO: MyClinicalOutcomes
NHS: National health service
OHS: Oxford hip score
OKS: Oxford knee score
PROM: Patient-reported outcome measures
