# Supplementary material for: Performance of EQ-5D, howRu and Oxford hip & knee scores in assessing the outcome of hip and knee replacements
Source: BMC Health Serv Res. 2016 Sep 22;16:512. doi: 10.1186/s12913-016-1759-x (PMC5034510; doi:10.1186/s12913-016-1759-x)
Supplement: Additional file 1: — Summary statistics scores using original scales. (DOCX 16 kb) [file 12913_2016_1759_MOESM1_ESM.docx]

Annex A

Summary statistics scores using original scales

| Original scores mean | | | | | | | |
| --- | --- | --- | --- | --- | --- | --- | --- |
|  | | **Pre-op score** | | **Post-op score** | **Change after surgery**  **(Post-op minus Pre-op)** | | |
| *NHS PROMs Hip Replacements (n=29,129)* | | | | | | | |
| OHS | 18.2 | | 38.5 | | | 20.2 |  |
| EQ-5D Index | 0.358 | | 0.772 | | | 0.415 |  |
| EQ-VAS | 65.4 | | 75.6 | | | 10.2 |  |
| *MCO Hip Replacements (n=74)* | | | | | | | |
| OHS | 20.0 | | 41.0 | | | 21.1 |  |
| *HowRu* | 6.6 | | 10.5 | | | 3.9 |  |
| *NHS PROMs Knee Replacements (n=29,907)* | | | | | | | |
| OKS | 18.9 | | 34.2 | | | 15.3 |  |
| EQ-5D Index | 0.409 | | 0.710 | | | 0.301 |  |
| EQ-VAS | 67.6 | | 72.2 | | | 4.6 |  |
| *MCO Knee Replacements (n=42)* | | | | | | | |
| OKS | 19.3 | | 36.8 | | | 17.5 |  |
| *HowRu* | 6.8 | | 9.9 | | | 3.1 |  |
